# Looking Upstream: Findings from Focus Groups on Public Perceptions of Source Water Quality in British Columbia, Canada

**DOI:** 10.1371/journal.pone.0141533

**Published:** 2015-11-05

**Authors:** Natalie Henrich, Bev Holmes, Natalie Prystajecky

**Affiliations:** 1 Center for Health Evaluation and Outcome Sciences, Providence Health Care Research Institute, Vancouver, British Columbia, Canada; 2 Michael Smith Foundation for Health Research, Vancouver, British Columbia, Canada; 3 BC Public Health Microbiology and Reference Laboratory, Vancouver, British Columbia, Canada; 4 Department of Pathology and Laboratory Medicine, University of British Columbia, Vancouver, British Columbia, Canada; NERC Centre for Ecology & Hydrology, UNITED KINGDOM

## Abstract

In association with the development of new microbial tests for source water quality (SWQ), focus groups with members of the public were conducted to gain insight into their perceptions of SWQ, behaviours and contaminants they think pose the greatest threat to its quality, and what/how they want to know about SWQ. Discussions revealed a low concern about SWQ in general, and in particular about microbial contamination. Participants identified behaviours that threaten SWQ, barriers to changing behaviour and suggestions for inducing change. A strong desire was expressed for water quality information to be interpreted and communicated in terms of how SWQ may impact human health and how their actions should be altered in response to test results. The information can be used to inform communication strategies and possibly impact policies associated with water quality testing and implementation of new tests. More broadly, awareness of the public’s understanding and beliefs about source water can be used in working with the public to adopt water-friendly behaviours, influence the content and methods of communicating with the public about water issues and water quality, and could contribute to the direction of future research and investment into water technologies to align with the public’s priorities.

## Introduction

New tests to assess microbial water quality hold the promise of improving public health and environmental health outcomes. Technological advances are allowing the development of new water quality tests that may be more sensitive and specific than currently available tests, with faster turn-around-times. However, little research has been performed to address the readiness of stakeholders and more specifically, the public, to accept novel water quality tests. Acceptance means different things depending on the type of stakeholder; in the case of the public, acceptance of water quality tests refers to their acceptance of the test *results*. Engaging with the public on new technologies (i.e., public engagement in science (PES)) may increase the likelihood that new technologies will be accepted as well as legitimizing the scientific and policy making process, thus potentially increasing trust in science and decision makers [1].

### Public Engagement in Science (PES)

Despite a long history of work on the diffusion of innovations [2], there is still a strongly-held belief that new and improved products and ways of doing things–especially when based on well-documented and accepted evidence—will naturally be incorporated into practice and policy [3]. Yet enabling the uptake of innovations such as new water quality testing requires opening “the black box of implementation” [4], which includes incorporating the views of those affected by the innovation. Frameworks for implementation contain many constructs relevant for examining the views of the public [4–6]. Addressing these constructs may, for the purposes of this study, contribute to the public’s acceptance of the results from new water quality tests and willingness to make alterations to their behaviours to reduce impact on water quality. These constructs explore factors such as the public’s needs with respect to water quality and water quality tests; barriers and facilitators to the public’s acceptance and use of test results; the alignment between existing policies, regulations and guidelines with uptake of test results; and the public’s access to the information and knowledge about water quality and the test results that they need to accept and use the results [4].

More broadly, working with the public in the pre-implementation phase is consistent with the growing movement for public engagement in science (PES), which calls for “upstream public engagement” [7] and the need for science to engage in dialogue with the public [8]. PES is recognized as valuable to the scientific process with public perspectives used to help guide the direction of scientific inquiry by identifying areas of concern and support among the public and using public input to inform science policy [1,9–14]. Feedback from the public is also seen as a key element in shaping knowledge translation to better meet the needs of the public. For example, studies consistently find that the public most trusts science information received from doctors or university scientists yet the primary sources of information are the internet and mainstream media, which are considered untrustworthy by much of the public [15–19]. Incorporating the public’s preferred sources of information into knowledge translation strategies can improve the public’s receptiveness to scientific advances.

In order for PES to be meaningful, “upstream activities should be linked back to the decision-making of scientists, industry and policy makers” [11: 642]. Focus groups are one of many methods are used to engage with the public and are useful because they “increase qualitative insights into specific topics, attitudes and behaviour, especially in fields about which people are not yet well informed and/or in which only limited social science research insights exist, and/or for which policy information is in an early stage and could benefit from citizen participation” [20: 343]. Some question whether it is necessary to engage the public during for all new technologies and claim that the public lacks the capacity to participate meaningfully on complex scientific issues. However, when PES is ineffective it is often because of insufficient or inadequate supports to enable successful participation or because the public’s input fails to reach decision makers [13]. ‘Failures’ in PES should be seen as opportunities to revise the process rather than as a reason to curb PES. Further, it cannot be known *a priori* when the public will be interested in engaging on an issue or the meaningfulness of their contributions. For scientists or decision makers to decide that PES is unnecessary for a given technological deliberation rests on an assumption that an understanding of the local or social context will not improve the development or implementation of a technology or that they already fully understand the public’s perspectives and concerns; these are unjust assumptions.

### Background on the study

Scientists at the British Columbia (BC) Public Health Microbiology Reference Laboratory (PHMRL), are using metagenomics to develop new tests to assess microbial water quality. The new water tests will be based on “metagenomic profiling” to identify novel markers of fecal contamination by looking for the presence of a wide range of bacteria, protozoa and viruses in watershed water. This approach may also be used to develop microbial source tracking (MST) tests, which can be used to determine the animal species that are the likely source of the microbial contaminants, such as geese, cows or humans. The new tests are being developed to characterize source water quality (SWQ) and, consequently, the new tests are relevant for assessing both the safety of drinking water as well as the health of ecosystems, along the source to tap spectrum.

To increase the likelihood that potentially transformative genomic technologies will be introduced into society, Genome BC (the project’s funder) requires a GE^3^LS (**g**enomics and its **e**thical, **e**nvironmental, **e**conomic, **l**egal and **s**ocial aspects) research component to be integrated into all of their funded projects [21]. As part of this GE^3^LS work, input was received from a wide-range of individuals who could potentially be users of the test or affected by the test results (see www.watersheddiscovery.ca). By using the stakeholder input to inform test characteristics, the information to be communicated to stakeholders about the test and its implications, and possibly policies and guidelines related to the use of the test and the results, uptake and receptiveness of the tests may be greater than if stakeholders had not been engaged during the test development phase.

Members of the public were one of the stakeholder groups from whom input was obtained.

The purpose of receiving input from the public was two-fold. First, having members of the public gathered to discuss water quality issues provided an opportunity to gain insight into the issues that concern the public about source water quality (SWQ), their level of concern, their perceptions of the kinds of behaviours that impact SWQ and which behaviours they would be willing to change, and their knowledge of where their drinking water comes from. Understanding the level of concern the public has about SWQ is important because it impacts how willing people would be to change their behaviours to improve water quality and how much support there may be for new research and investment into technologies for monitoring and improving water quality. Second, directly related to the new tests, scientists and knowledge translators wanted to understand any concerns or benefits that the public perceived as a consequence of the tests being used in source water where they live, the kind of information they want to know about the quality of the water and how they want to receive this information. These findings can be used to inform the communication strategy for disseminating information about the tests and sharing test results with the public, and could potentially shape present or future research related to water quality. Furthermore, it can assess the current knowledge about SWQ from the public and highlight educational needs and opportunities.

### Canadian public’s attitudes and knowledge about water quality

Some information about Canadians’ knowledge, attitudes and beliefs about water issues is already known from surveys. Canadians are generally not very concerned about the quality of their **drinking water** and **tap water quality** is perceived among the lowest risk hazards faced by the public [17,18,22,23]. Canadians are less concerned about tap water quality than Americans [24,25]; in contrast to Americans, of whom only 14% are unconcerned about the quality of their drinking water [26], 43% of Canadians perceive their drinking water as safe [27]. However, 83% of Canadians were concerned about the **quality of recreational water** (e.g., lakes in which they swim) and 68% thought lake water quality was deteriorating [28]. A survey in the town of Erickson, BC [17] found that residents felt microbial hazards were seen to pose only a moderate risk to drinking water, with risk perceived as lower if the source of microbial contamination was wildlife and higher if the source was treated human sewage or microbial contamination of water distribution lines. Chemical contamination from industrial pollution and resource development was seen to pose a greater threat to water safety than microbial contamination. This is in contrast to expert opinion (public health officials and other scientists) that sees the risk of microbial contaminants as high [17,29–31]. This misalignment in public vs. expert perspectives on the quality of water and the risks posed by different contaminants creates challenges in gaining public support for activities and research focused on reducing microbial contamination.

As governments and NGOs promote ways that the public can reduce their impact on source water, it will be helpful to know which behaviours the public recognize as a threat to water quality (and consequently where education may be required) and which behaviours they are willing to change. Eighty percent of Canadians admit to performing water-wasteful activities (e.g., 46% leave water on while brushing teeth) and between 15–20% report doing activities that negatively impact water quality (e.g., allow soapy water to run down a storm drain; hose down their driveway) [28]; although admitting they engage in these activities does not mean they are aware of these activities’ impact on water quality. In Vancouver, BC in 2010, 50% of residents reported flushing unused medicines or throwing them into the garbage (despite bans forbidding these methods of disposal), which is consistent with 51% of respondents reporting that they did not know that unused medicine should be returned to pharmacies [32]. Nation-wide, 72% of Canadians report flushing things down the toilet that could be disposed of other ways, including 16% who report flushing harmful or toxic materials [33].

Progress in changing public practices that affect water and increasing acceptance of technologies and investments for improving SWQ will be aided by a better understanding of why the public has a low overall level of concern about SWQ, which threats to SWQ are of greatest concern, which behaviours the public thinks pose a threat to water quality, what they perceive as barriers to changing their behaviours and what they report would make it easier to make changes. Although surveys have been carried out in Canada that address the public’s perceptions and use of water, little has been done qualitatively to delve deeper into why people think or act the way they do with respect to water. The focus groups provided an opportunity to explore these topics in depth and gain insight into the reasoning that underlies people’s beliefs and actions regarding water.

## Methods

Eight focus group sessions were conducted with the public. Six of the sessions were held in 3 BC communities (2 sessions/community), each of which was used as sampling site for the development of the new water quality tests. Two additional sessions were held with members of the public from anywhere in the greater Vancouver area who self-identified as being very knowledgeable about water issues or who were professionally associated with the water industry but who participated as members of the public rather than in a professional capacity (Table 1). These water knowledgeable people were included in the study because it was hypothesized that members of the public with expertise and passion for water may have different perspectives on SWQ issues than the general public and hence the focus groups began exploring if highly-informed publics held viewpoints that varied from less-informed publics.

**Table 1:** Watershed communities.

Community	Type and protection status of tap water source	Types of recreational water	Type of impact on recreational water	Source sampled for development of new water tests
1	Protected surface water	Fresh and coastal	Urban	Protected water for tap
2	Protected surface water	Fresh and coastal	Urban	Urban-impacted stream
3	Unprotected surface water (85% of popl’n); public and private groundwater (15% of popl’n)	Fresh	Agriculture	Agriculturally-impacted river
4	Protected surface water	Fresh and coastal	Urban	N/A

Led by experienced qualitative researchers, the participants were first introduced to the characteristics of the test under development. Specifically, they were informed of several reasons the new test would be more accurate than water quality tests that are currently used. The new tests:

- will create a water profile based on the identification of a range of microbial contaminants that indicate the presence of feces. Consequently, the new test is more likely to identify fecal contamination than tests that rely only on the presence of a single indicator, *Escherichia coli*.
- Will be preventative. The new test can identify a fecal pollution event as it is occurring by testing at the source and will identify contamination more quickly than tests that take place at the tap.
- May help identify the source of contamination. While it is easy to identify point-source pollution (e.g., pollution coming out of a pipe), it can be more difficult to locate non-point source (diffuse) pollution, such as from animals living in a watershed catchment area or from leaking septic tanks. Existing methods have no way of identifying which species are the sources of the pollution. Knowing the source of fecal pollution could help officials respond quickly to an event and, over the longer term, to help change practices to reduce the risk of future contaminations.
- Will focus on source water quality, which can be used for assessing both the safety of drinking water and ecosystem health.
- Will yield results faster than current tests with a processing time of approximately 4 hours rather than the current processing time for *E*. *coli* samples of 18–48 hours.

Next, the researchers led discussions addressing a set of semi-structured questions (Guide in S1 Focus Group Guide) including topics such as:

- the participants’ primary concerns about source water quality;
- what they believe impacts water quality (including the behaviours of people in their community);
- which behaviours they would be willing to change if they were shown to impact local water quality, and the barriers and facilitators to change;
- what information they would like to receive about water quality and how they would like to receive this information.

Focus group discussions were audio-recorded, transcribed and coded (in *QSR NVivo9*, a qualitative analysis program) using theme codes (overarching issues) developed based on the questions asked of participants and sub-theme codes (specific points that fit within an overarching theme) derived from the content of the discussions. Sessions from the same watershed were analysed together and are consequently presented together, as is also the case with the two “expert” groups. The results are framed within the 4 groups (protected watershed, urban watershed, agricultural watershed, experts). Themes were compared within and across focus groups to identify differences and similarities across watersheds, and were descriptively analysed. Focus group members provided written consent prior to participating and received payment for their participation. The study received ethics approval from the University of British Columbia’s behavioural review ethics board.

## Results

A total of 72 people participated in the focus groups. The participants ranged in age from 19 to 62, and there were approximately twice as many female participants than males (Table 2).

**Table 2:** Number of focus group participants, by community and sex.

Focus Group Community	Males	Females	Unknown	Total
1	7	12	0	19
2	8	12	0	20
3	6	12	0	18
4	2	12	1	15

In communities 1–3, the participants’ occupations were highly varied and included professionals (e.g., city planner, environmental scientist, architect), non-professionals (e.g., waitress, aerobics instructor, police officer), and non-workers (i.e., unemployed, stay-at-home moms, retired). Among the lay-experts (community 4), five were engineers or engineering students, six worked in water-related professions (e.g., water conservation education, water sanitation), and two were non-professionals with an interest in water (i.e., a water blogger and an artist with a passion for water). The demographics were similar across groups 1–3, and group 4 (“experts”) differed in that there were no non-workers.

The following describes the opinions expressed by participants in the focus groups. Where indicated, notable differences emerged between groups; otherwise, differences in opinions did not appear to correlate by group. As is standard in focus groups, the unit of analysis is the group rather than the individual and, consequently, analyses cannot be done to assess correlations between attitudes and specific demographic characteristics of group members. Given the demographic similarities across the focus groups, it was also not possible to explain differences between groups as a product of demographic characteristics (i.e., sex and age). Quotes from participants are included verbatim except where clarifications are required, as indicated by []. The results described below focus only on opinions/perspectives expressed by multiple participants.

### 1. Findings that could influence strategies to reduce public impact on SWQ and increase awareness about SWQ

#### Knowledge of source of drinking water

The majority of participants from communities 1–3 did not know the source of their drinking water, with no one in community 3 able to identify the source. Most of the experts were able to identify the sources of drinking water for the region.

#### Perception of SWQ

Overall, participants from all the watersheds thought they had very high quality drinking water. People described their water as “*the best water in the world*,” having a good smell and taste, and reported high confidence in its quality. As a participant explained, “*I’ve never really thought about it*. *We have such good water in Canada that it’s not really a concern*.*”* In one group, recreational water quality was seen as problematic because of geese feces in the water.

#### Importance of SWQ

SWQ was not an issue of high importance to most participants. Among those who were concerned about SWQ, one person in each of three of the four groups was concerned about SWQ because of the potential consequences of her children drinking unclean water. Three people mentioned concerns about SWQ from an environmental/ecological perspective, raising issues such as potential contamination from oil pipeline leaks, and the impact of climate change on water quality and quantity. Those who said that they were not concerned about SWQ explained that water quality is good so they don’t have to worry about it; one person said that as long as the treated tap water quality is good then SWQ doesn’t matter very much because it’s not essential to go swimming or boating, and several people explained that natural contamination (i.e., feces from wildlife) is supposed to be in the water because it is natural and hence not a cause of concern.

#### Threats to SWQ

Participants were asked to identify what they saw as the greatest threats to SWQ. The most common concern was **chemical contamination** with frequent reference to lawn chemicals and pesticides that get washed into storm drains. Other sources of chemical contamination mentioned include chemicals from agricultural and industrial activities, and oil spills (past spills as well as fears of future spills if the Northern Gateway pipeline is constructed). The threat of chemical contamination was often framed as a concern about run-off; primarily about activities that take place on the driveway or yard that end up in the drain (e.g., washing cars on driveway, powerwashing driveways). **Fecal contamination** was identified as a threat in each focus group although it was generally perceived to be less problematic than chemical contamination. It should be noted that the frequency with which fecal contamination was mentioned as a threat was likely influenced by the participants’ knowledge that the research project associated with the focus groups was working to improve identification of fecal contamination in source water. The source of fecal contamination that caused concerns varied across groups: In community 3, where the watershed is impacted by agricultural activity, participants mentioned run-off of manure as a threat to SWQ. In the other groups, participants identified wildlife (in particular geese), dogs, and sewage as sources of feces that could threaten SWQ. In each of the focus groups at least one person cited **overconsumption** of water as a primary threat to SWQ. Contamination of source water from **air pollution** was mentioned in three of the groups (e.g., car exhaust, contaminants from burning coal; mercury).

#### Public’s behaviours that impact SWQ

Participants identified behaviours engaged in by the public (as opposed to industry) that they believe affect SWQ. In two groups, the primary public behaviour seen to impact SWQ is the disposal of oils, solvents, paints and medicines down drains. In a third group, overuse of water and domestic pet waste (i.e, dog feces) were identified as the most damaging public behaviours to SWQ. Other behaviours listed by participants include washing cars and driveways, use of chemicals on lawns, dumping garbage and sewage into the water, and boating (fuel, sewage from houseboats).

#### What would facilitate changing behaviours that impact SWQ

In all four groups participants thought the best way to facilitate behaviour change is through **education and improving awareness**. They explained that people would be less likely to engage in activities that negatively impact water quality if they knew which activities did so.

*“I feel sometimes people don’t do stuff ‘cause they just don’t know*.*”*

*“I think there’s not enough awareness*. *Even the fact of taking prescription medication and putting them—flushing them down toilets*, *you know*, *it’s—you don’t hear of it a lot around here*. *But there is concern for that*, *you know*, *that it stays in the water for how long and it can’t go through the filtration system or it seeps through*. *So things like that*.*”*

Participants thought education should include explicit details about the health impacts of poor water quality and other consequences, such as the cost of treatment and the relative costs of keeping water clean versus cleaning dirty water (proactive vs. reactive measures). Suggested awareness-raising activities included marking storm drains to show that they empty into streams/oceans (such as the Trout Unlimited Canada's, *Yellow Fish* Road™). In three groups, participants thought that people would be more likely to reduce their impact on SWQ if they felt more connection to and appreciation for source water. They suggested that such connection and appreciation could be encouraged by promoting appreciation of our water and encouraging people to engage in activities that use recreational water. In one group, several suggestions were made involving government regulations that would prevent people from impacting SWQ by banning products and activities that are detrimental to SWQ.

#### What hinders changing behaviours that impact SWQ

Many barriers to behaviour change were described although none were mentioned by a large number of participants. In each group at least one person saw people’s laziness and lack of interest as preventing behaviour change. In two groups participants felt change was hindered by a lack of incentives to do the right thing (e.g., no financial rewards for using barrels to catch rain flow off of houses) and of consequences for doing the wrong thing (e.g., fines are not levied for watering lawns during periods of water restrictions or bans). In one group people felt strongly that the public should use environmentally-friendly products but expressed that these were not being used because they do not work well and that people will only be willing to use a “green” product if it does the job as well as a traditional product. In two groups, the cost of environmentally-friendly products was identified as a barrier to using these goods.

### 2. Findings that could influence development and communications about the new water quality tests

#### Importance of identifying fecal contamination in source water

Although fecal contamination as a threat to SWQ was mentioned in each group, the majority of participants were not concerned about this source of contamination. A recurrent explanation for the lack of concern was the belief that exposure to **feces does not pose a serious health threat**. People made comments about possibly getting an upset stomach but nothing serious, a lack of long term health consequences, and benefits of being exposed to fecal contaminants because it is unhealthy to live in an “*overly hygienic environment*.” Some shared the sentiment that there are more dangerous things in the water than feces (especially chemicals) so relatively speaking there isn’t reason to be concerned about the fecal contamination.

*“But I think in terms of sort of a lot of the general public out there*, *the idea of having poo in the water is a hysterical thing*. *Actually if you sit down and think about it*, *it’s not as bad as the rest of it*, *as the other options*.*”*

*“Well*, *chemical contaminants can do more harm to us than getting ill from exposure to sewage*.*”*

Others drew on personal experience and said they have gone swimming at beaches that were closed because of fecal contamination and they didn’t get sick and a belief that you just need to shower after swimming to avoid getting sick.

*“I’ve swum in [water X] every single year*, *and I love swimming in [water X]*. *And*, *you know*, *it’s not like you don’t pay attention when they say the fecal count is high or whatever*. *But you only live once*. *You can shower afterwards*. *It’s not like you’re guzzling it down by the litre*. *Just make sure you don’t put your head under*, *open your eyes under the water*. *But I would use it just the same*. *It’s [water X]*. *I mean*, *it doesn’t get much better than this*.*”*

More generally, one participant explained that there is always something bad in water but we’re all generally healthy (thus implying that the contaminants cannot be very bad for us). Some participants said they have not been concerned about health impacts of fecal contamination because they were not aware of any of the possible effects. In two groups, people distinguished between feces from wildlife, which they considered to be “natural,” and human feces. These participants expressed that things that are part of nature don’t pose a threat so we don’t need to be concerned about wildlife feces. On the other hand, they were concerned about water contaminated with human feces (sewage), which they believe has no place in source water.

*“And then the other issue is the natural things*. *I think goose poo is meant to be in water*, *and I think the dead leaves that are composting are meant to be in the water*, *and that’s probably one of the reasons it’s good for me in moderation*.*”*

Another reason that participants in two groups said they lacked concern about fecal contamination of source water was that drinking water is treated so contamination at the source doesn’t matter as long as the water coming out of the tap is clean.

For those participants who did have a high level of concern about fecal contamination of source water, the most common reason for concern was the possible **health effects**. Participants in each of the groups said that exposure to feces could make you very sick and recognized that diseases spread via feces; although, even among these participants there was often a belief that the health effects were not life threatening.

*“I’m just not sure*, *because I know in Third World countries and in disaster relief efforts*, *that the number one reason for death is not really the event*, *it’s the contamination of the water*. *Everybody gets a disease*. *Or not everybody*, *but a whole bunch of people get disease and die within the first week or two ‘cause there’s no fresh water*, *right*. *So water is really a concern when you get deep into it*. *And*, *like*, *diseases*, *a lot of diseases are spread through fecal matter*, *whether it be in water or on hands or anything*, *right…You don’t die*, *but you could have some health issues*, *I suppose*, *from that kind of thing*.*”*

In two groups, participants were concerned about fecal contamination of source water because it caused beach closures. Participants in two groups also felt that contamination in source water posed a threat to the quality of their drinking water because they did not trust the treatment process.

#### Potential impacts of water quality test results

Participants expressed both positive and negative perceptions of societal consequences of the new water quality tests revealing fecal contamination in water that previously had not been identified as contaminated. On the negative side, people may be scared to use/drink their water, farms could be damaged by stricter regulations or put out of business if they were identified as the source of contamination, water treatment facilities may undergo unnecessary expensive upgrades, and tourism could be hurt.

*“If you start to really get into the biological aspects of the water and potential risks to human health*, *then the message has to be conveyed effectively to the population*. *And that’s really hard to do*, *because if you say there’s elevated levels of anything*, *people are going to become afraid*.*”*

*“I wonder if they found that there was a lot more fecal matter in the water that the stricter rules and regulations surrounding farming around here would really hurt the industry*? *Because we rely on that really heavily*, *and then all of a sudden tighter rules*, *now people can’t maybe afford to do it in whatever manner is now necessary*. *And I wonder if that area would struggle then*.*”*

*“Oh*, *there goes tourism*.*”*

On the positive side, resources could be better used by accurately targeting the source of contamination and it could be the impetus for change to improve water quality: *“If you knew where it was coming from*, *you could fix the situation*.*”*

The general reaction among participants was that a test result showing the presence of feces-associated microbes would not lead them to use water any differently **unless** the identified microbes could be shown to be a health threat (pathogenic) and experts recommended that they change their behaviour (e.g., don’t swim in the water, boil water before drinking). People explained that the water would be of the same quality as before the test was conducted and they have not been getting sick; the only thing that would have changed would be their knowledge of what is in the water but if the water wasn’t causing health problems before the testing then there wouldn’t be a reason for the water to cause problems just because more was known about the water.

*“But I don’t know*, *if it was fine to drink yesterday—today we found something out and—it’s probably still fine to drink today*.*”*

*“I would just make sure that I knew what that information meant*. *If it doesn’t pose a health risk*, *it’s just kind of icky*. *That’s okay with me*.*”*

*“Without some perspective about what your information is providing*, *then this—it’s meaningless*, *right*? *Giving me information without telling me what the impact is and what the health impacts are is just pointless*. *It’s giving me—I don’t need more information*. *I need information in a way that makes me make good decisions about what’s going on*.*”*

In one group, participants said that there would be no reason to change their drinking water habits because the water is treated, so the presence of more microbes than had previously been detected would be irrelevant as the microbes are removed from the water before it reaches the tap. However, in another group several people said they would stop drinking tap water. In one group the response to the test results would depend on the source of the contamination; a commonly expressed view was that fecal contamination from humans or farm animals would cause them to change how they used the water but contamination from wild animals would not cause a behaviour change.

*“I think it might—this sounds weird*, *but what kind of feces it was*. *I feel like if—just the knowledge that it was human feces would really bother me*. *But if it was*, *like*, *beaver or something that’s been there forever*, *then it’s*, *like*, *well*, *this is part of the environment*.*”*

Participants said that if the tests revealed that water quality was better than previously believed there would be no effect on a societal level (except for one group that mentioned a possible boost to local tourism) and most said it would not affect their individual uses of water (although some would feel reassured about their current use of water). Across the groups, a few individuals said that a test finding better than expected water quality may lead them to drink tap water/stop filtering tap water and swim more in recreational bodies of water.

#### Information desired about SWQ

The main information participants want about SWQ is the meaning and implication of the test results. They do not want test scores; rather, people want the scores translated into information that is relevant for them in terms of assessing how they are to use source water and the risk associated with using the water. It was also suggested that the risk could be provided for different at-risk groups, such as young children and the elderly. For example, people don’t want to know the *E*. *coli* level at the beach but they do want to know if it is safe to swim.

*“I guess*, *just kind of agreeing [with others in the group] that I mean*, *for me*, *for my own personal water use*, *I’d just like to see some kind of scale*, *you know*. *Is it a good water-use day or a bad water-use day*? *And if it’s great*, *then I’ll go swimming*. *If it’s not*, *then maybe I’ll go—if it’s not quite as good*, *I can go canoeing*, *but not swimming*. *And then if it’s really hazardous—I don’t want to have to sit there and try and figure that out for myself*, *because I’m not an expert in these things*. *I’d just like to see some kind of scale*, *how good is it*.*”*

In two focus groups, participants felt that there is currently not enough information provided when contamination events occur and that they would like to have an improved alert system to notify the public of these events.

A minority of participants said that water quality information does not need to be shared with the public unless there is a problem. These people generally felt that *“no news is good news”* and that they only need to know about water quality if it poses a risk to their health.

#### Where information should be communicated

There was variability across groups in where they want water quality information communicated. Two groups had a strong preference for SWQ to be shown on the weather channel, similar to the way that air quality and the UV indices are shown. In one group people favoured accessing information from a reliable website (such as the city’s website), a sentiment echoed by a minority of individuals in two other groups. A few people in each of three groups prefer to get the information via mainstream media, although in one group participants clarified that they want the media to report information obtained from sources the public perceives as credible, preferring that the content originates from scientists rather than water suppliers. More broadly, in three groups a preference was voiced to receive information from professors and scientists, who are perceived as unbiased and knowledgeable, as opposed to people working in the industry who are seen as less trustworthy.

## Discussion

The focus groups yielded specific findings that would be beneficial to integrate into an implementation strategy about using SWQ test results to understand the status of source water and how human behaviours may impact SWQ. Drawing on implementation science, there are key constructs than we can draw on to interpret the focus group results [4–6]

1. It was found that the public’s needs are not being met when test results are difficult to locate and the results fail to be presented in a way that is meaningful and relevant to the public. People are less likely to use the results if they do not know how to interpret them; similarly Sofoulis [34] found that most Australian households found water meters incomprehensible and that it would be more useful to convey information about consumption with charts and guides corresponding to usage in different parts of the house (kitchen, bathroom, laundry, outdoors). As with water quality tests, the public would be more empowered to use water responsibly if they better understood the consequences of their actions. For implementation this would mean finding ways to share test results that align with the preferences of the public, such as the participants’ desire for test results that translate results into levels of risk and the corresponding actions that should be taken (e.g., safe to swim, swim with caution, don’t swim).
  The tests may also not be meeting the public’s needs because the public was more concerned about chemical contaminants than fecal contaminants. For implementation this will require educating the public about the importance of detecting fecal contamination (while not ignoring their concerns about chemical contamination).
2. Policies and regulations were not seen to support uptake of the test results or changes to behavior. Current Ministry of Health policies require that water quality test scores are posted on publicly accessible websites but do not require that the results are conveyed in a format that is meaningful to the public end users nor that the results are shared through public-friendly portals. Further, participants in the focus group identified a lack of incentive to change to more water-friendly behaviours because the government failed to provide incentives for positive behaviours (e.g., subsidies for purchasing containers to catch rain water) nor to enforce regulations like lawn watering restrictions (i.e., people were not getting fined for watering their lawn); a result also found among the public in Australia who wanted the government to help or provide incentives to make it more convenient to use water more efficiently [34]. People recognized a disconnect between their intentions for water-friendly behaviours and actually doing the behaviours and, at least some people, wanted the government to use ‘sticks and carrots’ to help push people in their communities from intention to action. This is disconnect between intention and action is consistent with theories showing that intention often does not translate into action unless many other internal, social and environmental conditions are present (for example, the role of normative beliefs on behaviors affecting the environment [35] and the Theory of Planned Behavior [36]). To support implementation, consideration should be given to enforcing and/or modifying regulations and policies to increase the likelihood that the public will use the test results and change their behaviours.
3. The public does not have adequate knowledge about the risks of fecal contamination in source water. Consequently, there will be a lack of motivation to access or use test results conveying information about fecal contamination or take steps to reduce or avoid fecal contamination in source water. The public cannot be expected to make informed decisions if they do not have the facts needed to make them informed. In this case, many people did not know that fecal contamination is a serious health threat; a fact which may lead to changes in attitudes and behaviors related to SWQ. Effective implementation will require increasing the public’s awareness about the facts and risks of fecal contamination. Additionally, for at least some members of the public there is a lack of awareness of how daily activities impact water quality and a step towards getting people to change their deleterious behaviours is to increase awareness that their behaviours are indeed affecting water quality. It is imperative that education is coupled with other strategies for changing public perceptions and behaviours related to SWQ. Education by itself is not effective for inducing behaviour change (consider, for example, people who smoke despite knowing the dangers of smoking and people who live a sedentary lifestyle despite knowing the toll it takes on their health). Drawing on social learning theory, outreach can be done in alignment with selecting appropriate models to deliver messages (e.g., use models who are seen by local communities as prestigious or successful; use models with the same sex/ethnicity as the target population) and using normative messaging (i.e., use the fact that people are biased to conform, or “copy the majority”) [37–39].

Among the focus group participants, low concern was expressed about SWQ. It could be argued that due to the public’s low level of interest for addressing SWQ in general and microbial contamination in particular (owing to their perception that quality is very good and that fecal contamination does not pose a serious health risk), that it isn’t necessary to engage the public about SWQ. Perhaps it would be appropriate for scientists, government and water suppliers to take the steps they believe are necessary for protecting source water while being open to public participation into decision yet without striving for a partnership with the public. At the very least, it may be adequate to have a pull, rather than a push, system for communicating with the public about water quality. In other words, instead of working to actively get information about water quality to the public (many of whom do not want it) it may be better to make the information readily available and allow those individuals who are interested in knowing about water quality to take the initiative to access the information, such as from a government website or signing up to receive text messages or emails. This approach would require that the public is made well aware of how to access the information and that the information is provided in a format that meets the public’s needs (such as translating test results into a scale of risk to human health). As Fischer explained, it is necessary to be mindful of when there is value in public participation [13] and this may be a situation in which the general public may not be motivated to engage. Rather, it may be more appropriate to engage with special interest groups and citizen organizations that are interested in this topic and can bring a non-expert perspective to the table.

There is a concern, though, with accepting the public’s contentment or complacency towards SWQ. Perceptions of water quality may be overly positive due to knowledge gaps and it may, in fact, be the responsibility of water or health officials to provide the public with the information they need to formulate informed opinions. Given that most participants in the focus groups were unaware of the source of their drinking water, it raises questions about how they can hold any opinion on the quality of their source water. Although these communities do usually have water of high quality, they have experienced adverse water quality events in the recent past (boil water advisories, waterborne outbreak and taste and odor issues) and it may be expected that these events would impact perceptions of water quality if the participants were aware of and understood the circumstances of these events. This is analogous to one of the core principles of PES, which is the need to provide public participants with the information and resources that would enable them to engage [9,13,20]. Taken more broadly, the public may become more interested and increasingly engaged in SWQ when they are provided information that addresses aspects of SWQ that matter to them and are provided this information in an accessible format.

Furthermore, if the public’s behaviours are impacting water quality then it becomes imperative to partner with the public to reduce these impacts. In this situation, it is necessary to use insights into why people behave in ways that are detrimental to SWQ, such as whether they engage in the behaviours because they don’t know they are damaging (as is the case for many people who improperly dispose of medications [32]) or if it is because of barriers to changing their behaviours (like the focus group participants’ dissatisfaction with the high cost and poor quality of green cleaning products). It also becomes necessary to raise awareness about risks associated with SWQ and, in particular, the dangers to human health associated with fecal contamination, as well as the benefits of taking a source to tap approach that can reduce pressures on water treatment by improving the quality of source water [40,41]. Unless the public fully appreciate the importance of good SWQ and have a realistic view of the current quality and risks to water safety it will be very difficult to motivate the public to actively make or support changes to improve SWQ.

Rather than reducing engagement with the public, acceptance and support for new water quality tests needs to be fostered among stakeholders and in particular, those stakeholders that see numerous downsides to the findings and few upsides. This engagement needs to extend beyond traditional stakeholder engagement that focuses on policy makers, subject matter experts and special interest groups to include all those affected by the new tests, including the public. Public engagement (communication, consultation and participation) is a commonly used approach in water management to gain the public’s trust [42–44]. In the ever-evolving field of genomics and metagenomics, much focus has been placed on ensuring that stakeholders are ready for new technologies, particularly the technologies that will be disruptive [45]. New water quality tests may be particularly disruptive to a wide-range of stakeholders if they identify water that was previously identified as ‘safe’ as now being ‘unsafe’. It is important to engage all stakeholders at all stages of test development, especially early stages of development, to not only promote the acceptance of the new test but to ensure that they are ready for the new test once it becomes available.

## Conclusions

Identifying how the public perceives SWQ, what they think poses threats to SWQ and their understanding of the relationship between their actions and SWQ is necessary for developing strategies to engage the public in doing or supporting actions to improve or maintain water quality. These strategies need to include communication or dialogue with the public that addresses knowledge gaps or misperceptions, as well as building on beliefs and behaviours that can be leveraged into actions that benefit water quality. Understanding what the public wants to know about water quality (and how they want to receive this information) is essential for communicating water quality information in general, and when reporting water quality test results, specifically. By incorporating the public’s preferences into policies and research agendas, a stronger partnership between water experts and the public can be fostered.

## Supporting Information

S1 Focus Group Guide (DOCX)
