# Supplementary material for: Looking Upstream: Findings from Focus Groups on Public Perceptions of Source Water Quality in British Columbia, Canada
Source: PLoS One. 2015 Nov 5;10(11):e0141533. doi: 10.1371/journal.pone.0141533 (PMC4634978; doi:10.1371/journal.pone.0141533)
Supplement: S1 Focus Group Guide — (DOCX) [file pone.0141533.s001.docx]

**S1. Focus Group Guide**

Focus Group Guide

Source water is the water from which our drinking water comes, before it is treated. It can be surface water, like rivers and lakes, or water pumped from the ground using wells. Surface water is treated before it makes it way to the tap.

Source water can be considered either protected or unprotected. Protected water sources in BC are protected from human activities so people can’t use or directly contaminate the water but these sources can still be impacted by natural source of pollution, like wildlife and erosion. Unprotected source water means the land surrounding the water (and the water itself) may be used for many purposes, such as recreational activities like swimming, boating, fishing and surfing, and industrial uses; human activities can impact source water, such as runoff from farms and logging, sewage leaks or disposal, and pesticides. Unprotected sources can also be impacted by wildlife such as bird, deer and beaver poop.

Are there any questions about these water definitions?

**Identification of Local Source Water**

1. Do you know the source of your drinking water?
   1. Do you know if your source water is protected or unprotected?
2. What are some of the main sources of water that are used for recreation in your community?

**Source Water Quality**

1. What do you think about the quality of source water in your community?
   1. Why? On what information are your perceptions based?
2. What do you see as the main threats to the quality of source water in your community?
3. What are your greatest concerns about source water in your community?
4. What behaviours or activities do you think impact the quality of your source water?
5. What information do you receive about the quality of water in your community? (Probe: Do you get information about source water quality and/or tap water quality?)
   1. When you get information about changes in water quality, how, if at all, do you alter the way you use water? (Probe: How, if at all, do you change how you use water for recreational activities?)
6. If a water quality test showed that the kinds of behaviours and activities that you do were contaminating your source water, which of your behaviours or activities would you change? How would you change them?
   1. What kind of challenges would you face in trying to change these behaviours or activities?
   2. What would make it easier for you to change these behaviours or activities?
7. What information do you want to know about source water quality?

**New Water Quality Test**

1. A new water quality test is being developed that will be better able to detect fecal contamination (poop from humans, wildlife and farm animals) in source water. Current tests only detect feces if E. coli is in the water. The new test will detect feces if any of about 20 different bacteria, viruses and protozoa are in the water. The new test will also be able to identify where the feces came from, such as cows, geese or human sewage.
   1. If the test showed that there are **more** feces in source water than we thought how, if at all, would this affect the way you use water?
   2. If the test showed that there are **less** feces in source water than we thought how, if at all, would this affect the way you use water?
2. If this new test was used to assess the quality of source water in your community, what impacts, good or bad, could there be to you, your community or local businesses and farms?

**Water Communication**

1. If there was a new technological development related to water, like the new test I’ve described or a new way to treat water, how would you prefer to hear about it?
2. If new information about water quality was available, how would you prefer to hear about it?
3. How important is it to you to receive information about water quality? About new water-related technologies?
